# Assessing the In Vitro Inhibitory Effects on Key Enzymes Linked to Type-2 Diabetes and Obesity and Protein Glycation by Phenolic Compounds of Lauraceae Plant Species Endemic to the Laurisilva Forest

**DOI:** 10.3390/molecules26072023

**Published:** 2021-04-01

**Authors:** Vítor Spínola, Paula C. Castilho

**Affiliations:** CQM—Centro de Química da Madeira, Universidade da Madeira, Campus da Penteada, 9020-105 Funchal, Portugal; vitor.spinola@staff.uma.pt

**Keywords:** type-2 diabetes, Lauraceae, polyphenols, digestive enzymes inhibition, aldose reductase inhibition, protein glycation inhibition

## Abstract

Methanolic leaf extracts of four Lauraceae species endemic to Laurisilva forest (*Apollonias barbujana*, *Laurus novocanariensis*, *Ocotea foetens* and *Persea indica*) were investigated for the first time for their potential to inhibit key enzymes linked to type-2 diabetes (α-amylase, α-glucosidase, aldose reductase) and obesity (pancreatic lipase), and protein glycation. Lauraceae extracts revealed significant inhibitory activities in all assays, altough with different ability between species. In general, *P. indica* showed the most promissing results. In the protein glycation assay, all analysed extracts displayed a stronger effect than a reference compound: aminoguanidine (AMG). The in vitro anti-diabetic, anti-obesity and anti-glycation activities of analysed extracts showed correlation with their flavonols and flavan-3-ols (in particular, proanthocyanins) contents. These Lauraceae species have the capacity to assist in adjuvant therapy of type-2 diabetes and associated complications, through modulation of the activity of key metabolic enzymes and prevention of advanced glycation end-products (AGEs) formation.

## 1. Introduction

Type-2 diabetes mellitus (T2DM) is a major category of diseases and is characterized by persistent elevated blood glucose levels (hyperglycaemia) resulting from absolute or relative deficiencies in insulin secretion and/or activity in tissues (insulin resistance) [1,2]. One therapeutic approach to decrease postprandial hyperglycaemia is to postpone digestion of carbohydrates through inhibition α-amylase and α-glucosidase in the small intestine [3,4,5]. Several carbohydrate-hydrolyzing inhibitor drugs (acarbose, voglibose, miglitiol) are clinically used, but present low efficiency and gastrointestinal side effects, such as abdominal bloating, cramping, increased flatulence, or diarrhea [6,7].

Obesity shows a high correlation with T2DM since most patients are either overweight or obese. In this sense, reducing dietary fats digestion is another way to prevent obesity, to stop weight gain and to slow down the rate of occurrence of T2DM [8]. Dietary fats are mainly hydrolyzed to monoglycerides and free fatty acids in the duodenum by the pancreatic lipase [9,10]. Orlistat^TM^ is the only pancreatic lipase inhibitor currently approved for long-term treatment of obesity. The most reported adverse effects include diarrhea, bloating and oily spotting, as well as hepatic adverse effects [10].

In this scenario, the plant kingdom emerges as a good source for searching for new effective hypoglycaemic and hyperlipidemic agents, such as phenolic compounds, with reduced side effects and that are cost-effective complements to synthetic drugs [5,6,7,11].

**Figure 1:**
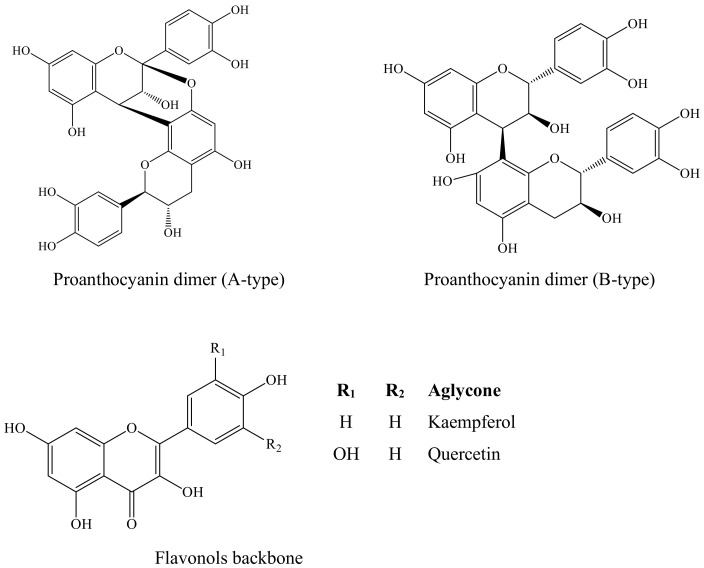
Structure of proanthocyanin dimers (A- and B-type linkages) and main flavonoids determined in the analysed Lauraceae extracts.

Some plants and herbs have a long history of traditional use in treatment of T2DM. [5,10,11,12]. The Lauraceae family comprises several plant species, some of high commercial value (including Laurus, Persea and Cinnamomum genera), due to their culinary, therapeutic, or industrial applications [13]. Cinnamon (*Cinnamomum* spp.) is one of the most popular spices used worldwide [14,15]. In vitro and in vivo data show that cinnamon improves post-prandial hyperglycaemia, triglycerides, LDL cholesterol, total cholesterol, and prevents protein glycation [10,15,16,17,18]. These health-beneficial effects are generally attributed to their high content of proanthocyanins (PACs), in particular those with A-type linkage [16,19,20]. PACs (or condensed tannins) consist of oligomers or polymers of monomeric flavan-3-ols (catechin/epicathecin units). PACs are mainly linked through C4–C8 or sometimes C4–C6 bonds, and these are termed as B-type. When an additional ether linkage is formed between C2–O7 or C2–O5, the compounds are called A-type PACs (Figure 1) [21,22].

In several studies, cinnamon showed high coumarin content, which may have hepatoxic effects (tolerable daily intake of 0.1 mg of coumarin/kg body weight) [20], so, in the present work, other Lauraceae, where coumarins were not detected, were studied as sources of PACs and other bioactive polyphenols.

**Figure 2:**
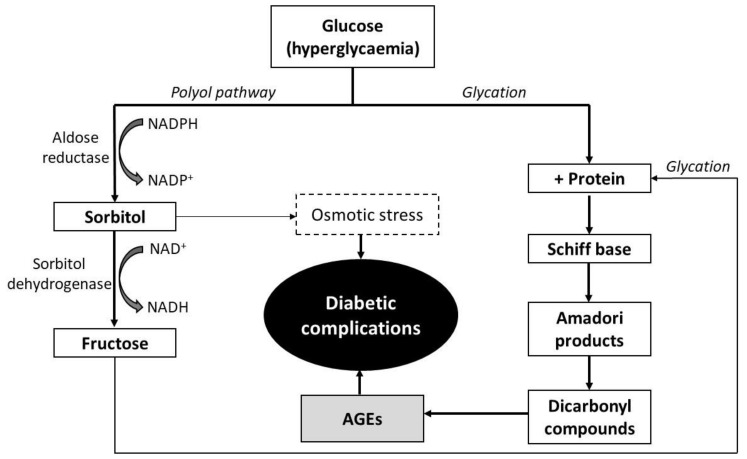
Direct link between the polyol rout and non-enzymatic protein glycation pathways leading to advanced glycation end-products (AGEs) formation. In hyperglycaemia conditions, excessive glucose is sequentially reduced to sorbitol and fructose by the combined action of human aldose reductase and sorbitol dehydrogenase. Accumulation of sorbitol in tissues is implicated in the development of degenerative complications of type-2 diabetes (neuropathy, nephropathy, retinopathy, etc.) due to osmotic stress. Fructose, the end-product of the polyol pathway, can be further conjugated with proteins increasing AGEs generation and related complications.

The Madeira (Madeira Island, Portugal) laurel forest, Laurisilva, is a subtropical forest which derives its name from the abundance and variety of Lauraceae species and other related endemisms, and is classified as UNESCO natural patrimony [23]. Previously [23], our research group reported PACs (A- and B-types) and flavonol glycosides as the main components of *Apollonias barbujana*, *Laurus novocanariensis* (laurel), *Ocotea foetens* and *Persea indica* (Figure 2), the most relevant Lauraceae species of Laurissilva. The anti-diabetic effects of similar plants (*Laurus nobilis*, *Persea americana* and *Ocotea bullata*) have been documented before [3,5,13,24,25,26,27,28,29,30], and similar properties were expected for targeted Lauraceae species. Therefore, this study was designed to evaluate for the first time the in vitro inhibitory effects of four Lauraceae plant extracts (*A. barbujana*, *L. novocanariensis*, *O. foetens* and *P. indica*) on digestive enzymes linked to the carbohydrates (α-amylase, and α-glucosidase) and lipids metabolism (pancreatic lipase), aldose reductase and protein glycation.

## 2. Results

Previously [23], the phenolic composition of the Lauraceae plants methanolic extracts was determined by HPLC-DAD-MS^n^ analysis. *A. barbujana* showed the highest total individual phenolic content (TIPC) and *P. indica* the lowest (Table 1). *A. barbujana*, *L. novocanariensis* and *O. foetens* were composed mainly by PACs (79.24–99.55%). *P. indica* presented the most diverse phenolic profile: flavonols (33.84%), flavan-3-ols (29.24%), phenolic acids (25.75%), flavanones (10.48%) and flavones (0.69%).

### 2.1. In Vitro Inhibition of Digestive Enzymes

*P. indica* was the most effective inhibitor of α-amylase, while *O. foetens* showed the lowest inhibitory activity towards this enzyme (Table 2). In the α-glucosidase assay, *P. indica* showed the strongest inhibition and *A. barbujana* the lowest (*p* < 0.05) (Table 1). *L. novocanariensis* (laurel) and *A. barbujana* were the most active samples towards pancreatic lipase, while *P. indica* showed the lowest effect (*p* < 0.05) (Table 2). In all assays, analyzed extracts displayed lower inhibitory activities than the positive controls, used in the form of pure substances. However, it must be taken into consideration that plant extracts are complex mixtures where the active substances are present in small amounts, so these activities are very relevant.

Since there are no published data regarding the inhibition of digestive enzymes by the targeted plants, a comparison with similar species will be further discussed. The peel, fruit, seed, leaf and pulp extracts of *P. americana* (avocado) strongly inhibited both α-glucosidase and α-amylase [5,25,26]. *Ocotea bullata* bark extracts demonstrated high inhibition of carbohydrate-metabolizing enzymes [24]. Different extracts of *L. nobilis* (bay laurel) inhibited α-glucosidase metabolic activity [3,13]. *L. nobilis* effectively inhibited α-amylase, but *Cinnamomum verum* (true cinnamon) extract was much more effective [31]. The same trend was observed for the inhibition of pancreatic lipase by *L. nobilis* and *C. verum* [11]. Additionally, the *C. verum* extract was a potent α-amylase inhibitor, while *L. nobilis* did not show anti-amylase activity. Both bark and leaf extracts of *C. verum* demonstrated inhibitory activity towards α-amylase (bark > leaf) [15]. *Cinnamomum osmophloeum* (indigenous cinnamon) twigs extract inhibited both α-glucosidase and α-amylase [20]. Studies with animals and human intervention trials are consistent with the effects demonstrated in vitro. Leaf extracts of *P. americana* reduced blood glucose levels and ameliorate hyperlipidemia in type-2 diabetic rats [12,32]. Consumption of *L. nobilis* leaf powder (in capsules or incorporated in cookies) improved glucose and lipid profile of diabetic patients [31] and induced a reduced glycemic response on healthy subjects [33]. Administration of *Cinnamomum cassia* (Chinese cinnamon) significantly reduced blood glucose levels and improved glucose tolerance on human trials [16,17]. In another study [10], subjects supplemented with *C. cassia* showed a significant decrease in serum lipase activity and lipid parameters and improvement of glycaemic targets.

The present data suggested that digestive enzymes inhibition is more influenced by specific phenolic types rather than the total amounts (TIPC) (r ≤ −0.657) (Table 3).

Plant extracts with abundant PACs appear to be suitable for the treatment of T2DM and obesity [22]. Since Lauraceae extracts (*A. barbujana*, *L. novocanariensis* and *O. foetens*) were composed mainly by PACs (79.24–99.55%) (Table 1), it was hypothesized that these compounds had an important role on the inhibition of digestive enzymes. Their inhibitory effect was more noticeable for the pancreatic lipase (Table 2), where extracts with high PACs content presented the best inhibitory effects (r = −0.874). Even with the lowest PACs composition (29.24%) (Table 1), *P. indica* showed better anti-hyperglycemic activity than other tested extracts (Table 2). This species presented the most complex phenolic composition (Table 1). Therefore, it is rationally presumed that other different active components contributed to this result. In the present work, the activity of carbohydrate-hydrolyzing enzymes was mainly affected by flavonols and flavan-3-ols/PACs (r ≥ −0.705) (Table 2). The catabolic activities of digestive enzymes are known to be affected by these classes of flavonoids. Previously [4,6,7], dicoumaroylated flavonol rhamnosides and quercetin/kaempferol glycosides were the main active compounds of *Machilus* spp. (Lauraceae) extracts against α-glucosidase. In another study [34], purified PACs from *M. philippinensis* strongly inhibited the activity of α-glucosidase. The strong anti-glucosidase activity of *C. burmannii* water extract was attributed to polymers of PACs and other flavan-3-ols [14]. PACs were the main inhibitors of *C. osmophloeum* extract against carbohydrate-hydrolyzing enzymes [20]. The peel extract of *P. americana* (high in epicatechin and galloylated derivatives) displayed the highest anti-amylase activity [5]. It seems that the higher the degree of polymerization of PACs, the better the inhibitory activity towards enzymes of the carbohydrates and lipids metabolism [21,22,35]. Besides molecular size, the structure of PACs seems also determinant to contribute to a more effective inhibition of pancreatic lipase. The A-type bonded polymeric PACs are found to be more potent to suppress pancreatic lipase activity than the higher abundant B-type PACs [21]. This agrees with the present data (Table 2), where species with A-type PACs (*A. barbujanas*, *L. novocanariensis*, and *O. foetens*) (Table 1) displayed higher inhibitory activities towards pancreatic lipase (r = 0.904).

### 2.2. In Vitro Inhibition of Human Aldose Reductase

For the aldose reductase assay, *P. indica* was the most active sample, while *O. foetens* showed the lowest inhibitory activity (*p* < 0.05) (Table 2). In this study, flavonols seem the main inhibitors of human aldose reductase (r = −0.795) (Table 3).

Chronic hyperglycemia is fundamental for the development and progression of diabetic micro- and macrovascular complications through various hyperglycemia-induced metabolic derangements, namely increased polyol pathway flux and increased advanced glycation end-products (AGEs) formation [28,36]. Aldose reductase is the first enzyme of the polyol pathway that catalyzes reduction of glucose to sorbitol [27] (Figure 2).

In normal conditions, aldose reductase has low substrate affinity to glucose, so that the conversion of glucose to sorbitol is little catalyzed. However, in chronic hyperglycaemia, the increased availability of glucose leads to the increased activity of the polyol pathway. Sorbitol does not readily diffuse across cell membranes and its intracellular accumulation is implicated in the pathogenesis of some diabetic complications (cataract, neuropathy, and retinopathy) [25,34,35]. This can be efficiently averted by inhibition of key enzyme aldose reductase [24]. Thus, the development of aldose reductase inhibitors might provide a therapeutic approach to prevent or delay progression of some diabetes complications [25,34]. Plant-derived extracts or phytochemicals are potential alternatives to synthetic inhibitors (Sorbinil^®^, Tolrestat^®^, and Epalrestat^®^) against aldose reductase that present limited efficiency or undesirable effects [24,25,34].

In a previous study [27], a methanolic leaf extract of *P. americana* strongly inhibited aldose reductase. A *L. nobilis* extract showed moderate inhibitory activity towards this enzyme [28]. Flavonoids (kaempferol glycosides, epicatechin and myricitrin) isolated *from Litsea japonica* leaves (another Lauraceae species) exhibited considerable inhibition of aldose reductase [36]. Considering that catechin is the base unit of PACs (found in high amounts in the analyzed extracts: 29.24–99.55%) (Table 1), the inhibitory potential of a (+)-catechin standard was further evaluated in detail. This molecule showed high inhibitory activity towards aldose reductase, but lower than quercetin standard (Table 2). The same pattern has been described previously, where the inhibitory activities of flavan-3-ols were weaker than flavonol-type compounds [18,36]. The present data (Table 2) corroborate this hypothesis, since *P. indica* (with the highest flavonol composition) was the most active sample.

### 2.3. In Vitro Inhibition of Ribose Mediated BSA-Glycation

Lauraceae leaf extracts showed relevant anti-glycation effects (IC50 ≤ 2.42 mg mL^−1^ DE) (Table 2). *P. indica* and *L. novocanariensis* displayed the highest inhibitory activities, while *O. foetens* the lowest (*p* < 0.05). All extracts were more effective to prevent BSA glycation than aminoguanidine (AMG) (*p* < 0.05). Aminoguanidine (Pimagedine) was developed as a drug for kidney diseases, namely for the treatment of diabetic nephropathy, due to its ability to prevent the formation of AGEs [35]. However, clinical trials found increased risk of side effects and AMG was discontinued, and, nowadays, it is used only as an investigational agent [18,37]. One justification for this result is that AMG does not inhibit the initial stage of protein glycation (Schiff base and Amadori products formation) [38]. It is a nucleophilic agent that scavenges reactive carbonyl intermediates to form relatively nontoxic adducts, thus preventing their conversion to AGEs.

Another consequence of the elevated blood glucose is an increase in non-enzymatic glycation of proteins such as hemoglobin A1c (HbA1c) and serum albumin [1]. Protein glycation, also known as Maillard reaction, occurs when the carbonyl group of a reducing carbohydrate reacts with an amino group of proteins to form a highly reversible Schiff base intermediate, which rearranges to form a more stable Amadori product (early phase) (Figure 2) [30,35,39]. Then, Schiff bases or Amadori products go through a complex series of reactions (rearrangement, oxidation, dehydration, polymerization) to generate reactive dicarbonyl compounds, including methylglyoxal, glyoxal and 3-deoxyglucusone (middle phase), which further form adducts with proteins to generate AGEs (late phase) [15,38,40].

These harmful compounds are important pathogenic mediators in diabetes-related complications, through a series of pathological changes in the structure and function of extracellular matrix proteins and lipids [15,19]. These ailments include nephropathy, neuropathy, retinopathy, coronary heart disease and atherosclerosis [18,29,35]. Hence, inhibition of AGEs formation has been frequently considered as an efficient target to slow down the progression of some diabetic complications [18,30,41]. To avoid the problems related to the synthetic molecules (like AMG), research has been directed to phytochemicals, such as phenolic compounds, which have proven effective anti-glycation properties with a reduced risk of adverse effects [18,30,41].

In the present work, the inhibition of AGEs formation was poorly correlated with TIPC (r = −0.530), suggesting that the type of phenolic compounds was more important to the prevention of protein glycation than the total amounts. Flavonols (r = −0.735) seem to contribute the most for the obtained results (Table 3). Quercetin standard showed the strongest inhibitory activity (Table 2), and it has been suggested as a finer substitute to AMG due to its potent anti-glycation effects [42].

Previously [29,30,39], different extracts of *L. nobilis* prevented the formation of AGEs. Gallic acid was the main compound in the extract and its anti-glycation effect was partially attributed to this phenolic [30]. Gallic acid was absent in *L. novocanariensis* extract [23], therefore, we hypothesize that the activity of this species can be attributed to PACs. Bark extracts of *C. verum* showed significantly high antiglycation activity than leaf extracts [15]. Bark extracts had higher total PACs content than leaf counterparts. Catechin, epi-catechin, and procyanidin B2 significantly inhibited AGEs formation and are the main anti-glycation components of *Cinnamomum* spp. extracts [18,19]. Their AGEs-inhibitory effects are attributed to both their antioxidant activities and capacity to scavenge reactive carbonyl species.

The observed anti-glycation activities (Table 2) are attributed to different phenolic compositions in the various extracts. In the case of *P. indica*, the variety of phenolic compounds seem to act synergistically to inhibit AGEs formation. Flavonoids (kaempferol glycosides, epicatechin and myricitrin) from *L. japonica* strongly inhibited the formation of AGEs [35]. Previous works [38,40] have shown that the flavonoids-PACs mixture improves the anti-glycation activity of extracts. Based on Table 2, quercetin was more active against BSA-glycation than catechin. Flavan-3-nols (where catechin and PACs are included) seem lesser effective inhibitors of protein glycation than flavonols. Catechin has the same number of hydroxyl groups in the same positions as quercetin but lacks the 4-oxo function and the 2,3-double bond (Figure 1), which contributes to electron delocalization, stabilizes the phenoxy radical and results in higher relative anti-glycation potential [41]. This partially justifies the obtained results (Table 2), since extracts with higher PACs content (*A. barbujana*, *L. novocanariensis* and *O. foetens*) displayed lower inhibitory activities than *P. indica*. This pattern was consistent with the inhibition of aldose reductase, supported with a strong correlation (r = 0.999) between these two assays. In fact, there is a close link between AGEs generation and the overactivity of aldose reductase (Figure 2). Fructose, the end-product of the polyol pathway, acts as precursor/activator of AGEs, being more reactive than glucose as glycating agent (approximately 10 times) due to a faster conversion of its Amadori products [40]. Hence, inhibition of aldose reductase activity also contributes to limit protein glycation and AGEs accumulation. The analyzed extracts exerted a double action i.e., via simultaneous inhibition of aldose reductase and protein glycation, suggesting another potential therapeutic approach against hyperglycaemia-induced complications besides modulation of digestive enzymes activity.

## 3. Materials and Methods

### 3.1. Chemicals and Reagents

d-(−)-fructose, soluble starch (p.a.), sodium azide (> 99%), potassium iodate (99.5%) were acquired from Merck (Darmstadt, Germany). Acarbose, aminoguanidine hydrochloride (AMG, ≥ 98%), bovine serum albumin (BSA, ≥ 98%), intestinal acetone powder from rat source of α-glucosidase, α-amylase from porcine pancreas (type VI-B), lipase (type II; from porcine pancreas), dl-glyceraldehyde (≥ 98%), β-mercaptoethanol (≥ 99%), sodium carbonate (100%), p-nitrophenyl-α-d-glucopyranoside (α-pNPG), *p*-nitrophenyl butyrate (pNPB), orlistat and d-(−)-ribose (≥ 99%) were acquired from Sigma-Aldrich (St. Louis, MO, USA). Human aldose reductase (HAR) was purchased from Prozomix (Northumberland, UK) and β-nicotinamide adenine dinucleotide reduced tetrasodium salt hydrate (NADPH, ≥ 97%) from Calbiochem (San Diego, CA, USA). 1-Deoxynojirimycin (1-DNJ; 95–99%) was obtained from Biopurify phytochemicals LTD (Chengdu, China). (+)-Catechin hydrated (> 99%) and quercetin dihydrate (> 99%) were acquired from Extrasynthese (Genay, France) and Riedel-de Haen, respectively.

### 3.2. Sample Preparation and Extraction of Phenolic Compounds

Leaves from four different Lauraceae species (*A. barbujana*, *L. novocanariensis*, *P. indica* and *O. foetens*) endemic from Laurissilva florest were collected in 2013 at Ribeiro Frio (Madeira Island, Portugal). Temperatures in that area of Madeira Island varied between 15 to 17 °C and the average precipitation was around 24 mm by the time of leaf harvest. The methanolic extracts preparation was described in detail previously [23]. Briefly, the lyophilized material was mixed with methanol (1:25 solid material:solvent ratio), ultrasonicated (60 min), filtered (Whatman No.1 filter papers) and concentrated to dryness under reduced pressure in a rotary evaporator (at 40 °C) (Buchi Rotavapor R-114, Flawil, Switzerland). The resulting dry extracts were stored at 4 °C until further analysis.

### 3.3. In Vitro Anti-Diabetic and Anti-Obesity Assays

#### 3.3.1. α-Amylase Inhibition Assay 

The assay was performed as described before [43]: 20 µL of sample extract (serial dilutions) and 40 μL of 2 g L starch solution were mixed with 20 μL of α-amylase (0.1 mg mL^−1^). All solutions were prepared in 0.1 M phosphate buffer (pH 6.9). After incubation (20 min; 37 °C), the reaction was stopped by the addition of 80 μL of 0.4 M HCl followed by 100 μL of 5 mmol L^−1^ I2 (in 5 mmol L^−1^ KI) and the absorbance was read at 620 nm (Victor3 microtiter reader, PerkinElmer, Waltham, MA, USA). Acarbose was used as positive control. Inhibition (% I) of α-amylase activity was calculated using the following formula:

% I = [((A_C_ − A_CB_) − (A_S_ − A_SB_)) / ((A_C_ − A_CB_))] × 100 (1)

where A_C_, A_CB_, A_S_, and A_SB_ are the absorbance of control, control blank, sample and sample blank, respectively. Control of the experiment contains all the reagents except extracts, whereas sample blanks were without the enzyme. The IC_50_ values (mg mL^−1^ of dry extract or reference compound) were determined from the least-squares regression line of the logarithmic concentrations plotted against percentage inhibition. This value corresponds to the concentration of the extracts able to reduce the enzyme activity by 50% with reference to the control.

#### 3.3.2. Rat α-Glucosidase Inhibition Assay

For this assay, a previous protocol was used [43]: 0.5 g of intestinal acetone powder from rat was dissolved in 10 mL of 0.1 M potassium phosphate buffer (pH 6.9) and sonicated for 10 min. After centrifugation (Sigma 3K30, C&M Scientific Ltd, Livingston, UK) at 1753 g for 10 min at 4 °C, the resulting supernatant was diluted 5 times with the above buffer and was used as the enzyme solution. Then, in a 96-well plate, 50 μL of sample extract (sequential dilutions) were combined with 50 μL of enzyme solution (0.1 mg mL^−1^) and 50 μL of 5 mmol L^−1^ α-pNPG solution. All solutions were prepared in 0.1 M phosphate buffer (pH 6.9). The mixture was incubated at 37 °C for 20 min in the dark. Finally, 100 μL of 0.1 M Na_2_CO_3_ solution were added and the absorbance was read at 405 nm (Victor3 microtiter reader, PerkinElmer). Acarbose and deoxynojirimycin (1-DNJ) were used as positive controls and the obtained inhibitory activities were expressed as the IC_50_ value (mg mL^−1^ of dry extract or reference compound) (calculated as explained in the previous section).

#### 3.3.3. Pancreatic Lipase Inhibition Assay

The methodology for this assay was previously published [43]. Briefly, 40 µL of sample extract (serial dilutions) were mixed with 20 µL of substrate solution (10 mM of *p*-NPB in ethanol) and 40 µL of the enzyme (2.5 mg mL^−1^ in 0.1 M phosphate buffer, pH 8.0). After incubation (20 min; 37 °C) absorbance was read at 405 nm (Victor3 microtiter reader, PerkinElmer). Orlistat was used as positive control and the obtained inhibitory activities were expressed as the IC_50_ value (mg mL^−1^ of dry extract or reference compound) (calculated as explained in Section 3.3.1).

#### 3.3.4. Aldose Reductase Inhibition Assay

The inhibition of human aldose reductase (HAR) activity was measured as follows [43]: in a 96 well-plate (UV-transparent), 25 µL of extract solution (serial dilutions) were mixed with 25 µL of 10 mM dl-glyceraldehyde and 25 µL of enzyme solution (1 mg mL^−1^). All solutions were prepared in 0.1 M phosphate buffer (pH 6.2) containing 0.2 mM ammonium sulfate and 5 mM β-mercaptoethanol. The reaction was initiated with the addition of 50 µL of 0.5 mM NADPH solution and incubation at 37 °C for 20 min. The decrease in the absorption of NADPH was measured at 340 nm (Victor3 1420 microtiter reader, PerkinElmer) over 0 and 20 min of reaction. Quercetin and (+)-catechin standards were used as positive controls. The obtained inhibitory activities were expressed as the IC_50_ value (mg mL^−1^ of dry extract or reference compound) (calculated as explained in Section 3.3.1).

#### 3.3.5. BSA Glycation Inhibition Assay

The inhibition of protein glycation followed a previous protocol [43]. In brief, 100 μL of BSA solution (10 mg mL^−1^), 100 μL of ribose solution (0.5 M) and 40 μL of sample extracts (serial dilutions) were combined in a black 96 well-plate. All solutions were prepared in 0.1 M potassium phosphate buffer (pH = 7.4). After incubation (24 h at 37 °C), plates were analysed at an excitation wavelength of 355 nm and emission wavelength of 460 nm (Victor3 microtiter reader, PerkinElmer). Aminoguanidine (AMG) and quercetin and (+)-catechin standards were used as positive controls. Inhibition (% I) of BSA-glycation was calculated using the following formula:

% I = [((F_C_ − F_CB_) − (F_S_ − F_SB_)) / ((F_C_ − F_CB_))] × 100 (2)

where F_C_, F_CB_, F_S_, and F_SB_ are the fluorescence of control, control blank, sample and sample blank, respectively. Control of the experiment contains all the reagents except extracts, whereas sample blanks were without ribose. The IC_50_ values (mg mL^−1^ of dry extract or reference compound) were determined from the least-squares regression line of the logarithmic concentrations plotted against percentage inhibition. This value corresponds to the concentration of the extracts able to reduce the protein glycation by 50% with reference to the control.

### 3.4. Statistical Analysis

All samples were assayed in triplicate and results are given as the means ± standard deviations. Analysis was carried out by means of a one-way ANOVA with Tukey’s post-hoc test using SPSS for Windows, IBM SPSS Statistics 20 (SPSS, Inc., Chicago, IL, USA). Pearson correlation coefficients (r) were determined to corroborate relationships between selected parameters.

## 4. Conclusions

In this work, four different Lauraceae plant extracts inhibited key enzymes linked to T2DM and obesity. Additionally, the analyzed extracts were stronger inhibitors towards BSA-glycation than the reference compound AMG. In general, *P. indica* exhibited the best inhibitory potencies in all assays; *L. novocanariensis* showed the most effective anti-lipase effect. Hence, these two species may constitute a promising functional food material for suppressing sugars and lipids uptake and prevent hyperglycaemia-associated complications. The different phenolic composition of extracts dictated the obtained results. PACs and flavonols glycosides were important contributors to the obtained data. The absorption of PACs oligomers is known to be very low [34]. Therefore, it is believed that the main anti-diabetic activities of these compounds are due to the inhibition of digestive enzymes in the gastrointestinal tract. Considering the in vivo data for similar species (*P. americana* and *L. nobilis*) [12,31,32,33], it is extrapolated that these extracts would be effective hypoglycaemic and hypolipidemic agents. However, additional biological tests are necessary to fully evaluate their therapeutic efficiency.

## Figures and Tables

**Table 1:** Total individual phenolic content (TIPC) (mg g^−1^ dry extract) of Lauraceae leaf methanolic extracts determined by HPLC-DAD (data from [23]).

	Phenolic Acids	Flavones	Flavan-3-ols	Flavones	Flavanones	TIPC	Extraction Yield (%)
*A. barbujana*	Not detected	15.56 ^c^	72.82 ^d^	0.60 ^d^	Not detected	88.98 ^d^	4.62
*L. novocanariensis*	0.5 ^a^	2.69 ^a^	64.59 ^c^	0.23 ^c^	Not detected	68.01 ^c^	4.68
*O. foetens*	Not detected	Not detected	18.35 ^b^	0.09 ^a^	Not detected	18.44 ^a^	4.48
*P. indica*	5.6 ^b^	7.36 ^b^	6.36 ^a^	0.15 ^b^	2.28	21.75 ^b^	9.84

Means in the same column not sharing the same letter are significantly different at the *p* < 0.05 probability level.

**Table 2:** In vitro inhibitory activities of Lauraceae leaf extracts towards carbohydrate-hydrolyzing enzymes, pancreatic lipase, aldose reductase and BSA-glycation. Results are expressed as the IC_50_ value (mg mL^−1^ of dry extract or pure compound). Acarbose, 1-Deoxynojirimycin, Orlistat, Quercetin, (+)-Catechin and Aminoguanidine were used as positive controls for α-amylase, α-glucosidase, lipase, and aldose reductase and BSA glycation assays, respectively.

	α-Amylase	α-Glucosidase	Lipase	Aldose Reductase	BSA Glycation
*A. barbujana*	1.15 ± 0.05 ^d^	5.54 ± 0.20 ^f^	2.60 ± 0.12 ^b^	0.38 ± 0.01 ^c^	1.15 ± 0.03 ^d^
*L. novocanariensis*	0.57 ± 0.02 ^c^	4.08 ± 0.20 ^e^	2.40 ± 0.10 ^b^	0.37 ± 0.02 ^c^	1.06 ± 0.04 ^c^
*O. foetens*	1.52 ± 0.03 ^e^	3.59 ± 0.12 ^d^	3.69 ± 0.08 ^c^	1.02 ± 0.05 ^e^	2.42 ± 0.16 ^e^
*P. indica*	0.50 ± 0.02 ^b^	2.83 ± 0.09 ^c^	4.70 ± 0.12 ^d^	0.28 ± 0.01 ^b^	0.96 ± 0.04 ^c^
Acarbose	0.02 ± 0.001 ^a^	0.12 ± 0.01 ^b^	−	−	−
1-Deoxynojirimycin	−	0.01 ± 0.01 ^a^	−	−	−
Orlistat	−	−	0.47 ± 0.02 ^a^	−	−
Quercetin	−	−	−	0.10 ± 0.01 ^a^	0.11 ± 0.01 ^a^
(+)-Catechin	−	−	−	0.76 ± 0.04 ^d^	0.24 ± 0.02 ^b^
Aminoguanidine	−	−	−	−	9.56 ± 0.36 ^f^

Means in the same column not sharing the same letter are significantly different at *p* < 0.05 probability level. Data represent the mean ± standard deviation (*n* = 3).

**Table 3:** Correlation coefficients (r) observed among phenolic composition of Lauraceae leaf extracts and evaluated in vitro bioactivities.

Parameters	α-Amylase	α-Glucosidase	Lipase	Aldose Reductase	BSA Glycation
TIPC	−0.657	−0.621	−0.269	−0.482	−0.497
Phenolic acids	−0.020	−0.310	+0.839	−0.492	−0.467
Flavonols	−0.792	−0.756	−0.740	−0.761	−0.735
Flavan-3-ols/PACs	−0.773	−0.705	−0.874	−0.325	−0.350
Flavones	+0.097	+0.917	−0.367	−0.433	−0.424
Flavanones	−0.296	+0.464	+0.870	−0.222	−0.254

TIPC: Total Individual Phenolic Content (sum of the phenolic contents determined by HPLC-DAD in [23]). PACs: proanthocyanins. BSA: bovine serum albumin.

## Data Availability

Data are contained within the article.

## Sample Availability

Samples of the tested extracts are available from the authors.
